# Effects of hypoxia on the olfactory sensitivity of gilt-head seabream (*Sparus aurata*)

**DOI:** 10.1242/jeb.249771

**Published:** 2025-01-09

**Authors:** Liam R. Tigert, Peter C. Hubbard, Cosima S. Porteus

**Affiliations:** ^1^ Department of Biological Sciences, University of Toronto Scarborough, Toronto, ON M1C 1A4, Canada; ^2^ Cells and Systems Biology, University of Toronto, ON M5S 1A1, Canada; ^3^ CCMAR-CIMAR Laboratório Associado, Universidade do Algarve, Campus de Gambelas, 8005-139 Faro, Portugal

**Keywords:** Environmental physiology, Olfaction, Sensory physiology, Electro-olfactogram, Anthropogenic stressor

## Abstract

Coastal environments around the world are becoming increasingly hypoxic owing to anthropogenic effects. We hypothesized that, because the olfactory epithelium is in contact with the external environment, decreased external oxygen will impair olfaction. We performed electro-olfactograms on juvenile gilt-head seabream (*Sparus aurata*) and measured the response to three amino acids at five different concentrations (1×10^−7^ to 1×10^−3^ mol l^−1^) in normoxic (20 kPa O_2_) and two hypoxic conditions (12.5 and 5.7 kPa O_2_). For the first time, we show that both mild and moderate hypoxia decreased the olfactory response to two out of three odorants. As more coastal areas become hypoxic, it is important to understand how hypoxia may impair the sensory systems of fishes, which can have individual- and population-level effects and important implications for our food supply.

## Teaser

**Highlighted Article:** Electrophysiology shows that low external oxygen levels can impair the olfactory sensitivity of a coastal marine fish species.

## INTRODUCTION

Olfaction is used by fish for many different aspects of life history, such as foraging for food (Giaquinto and Hoffmann, 2010; Johannesen et al., 2012; Sola et al., 1993), finding suitable habitats (Miller-Sims et al., 2011; Mitamura et al., 2005), intraspecific communication and finding mates (Bhatt et al., 2002; Coleman and Rosenthal, 2006; Nikonov and Maruska, 2019), and avoiding predators and contaminants in the environment (Arvigo et al., 2019; Hidaka and Tatsukawa, 1989; Miyai et al., 2016; Vilhunen and Hirvonen, 2003). Olfaction is advantageous over other senses because fish can detect things in a visually complex environment, where the line of sight may be broken, or in murky waters where vision is limited (Hara, 1976; Laberge and Hara, 2001), such as in coastal environments. However, coastal environments around the globe are experiencing a decrease in oxygen, mainly driven by anthropogenic activities, such as eutrophication, and contaminants entering the water (Breitburg et al., 2018; Vollenweider, 1992). In some locations, the oxygen has decreased to below 2 mg l^−1^ O_2_, and these coastal hypoxic zones are becoming more and more common (Diaz and Breitburg, 2009; Vollenweider, 1992). Therefore, if hypoxic conditions impair olfaction in fish, it may limit the ability of fish to detect important odorant sources, and negatively impact their fitness.

Fish employ a set of responses to deal with both short-term and chronic hypoxia exposure to restore internal oxygen levels if the oxygen partial pressure is above their critical oxygen tension (*P*_crit_), the point in which the oxygen in the environment is too low for normoxemia (normal oxygen tension in the blood) to be achieved (Farrell and Richards, 2009; Jonz et al., 2016; Mandic et al., 2008). To achieve this, fish will increase their ventilatory rate, undergo bradycardia (Perry et al., 2009), and increase the oxygen-binding affinity of haemoglobin (Smit and Hattingh, 1981). In long-term exposure to hypoxia, fish may also remodel their gills to increase the surface area for greater oxygen extraction from the water (Soor et al., 2024). Fish may also employ different behavioural responses to avoid hypoxic conditions, such as aquatic surface respiration (Kramer and McClure, 1982), or leaving the hypoxic environment all together (Tigert et al., 2022). Therefore, fish can maintain normoxemia, even if the external environment is hypoxic. As such, it is quite common for external organs, such as the eyes, gills and the olfactory epithelia, to be exposed to hypoxic conditions while the fish remains normoxemic.

Cells that are highly sensitive to oxygen deprivation include neurons, but it is currently unknown whether sublethal levels of hypoxia can negatively impair olfaction in fishes (Tigert and Porteus, 2023). For example, sturgeon held in hypoxic conditions (15% oxygen saturation) experienced apoptosis in the nervous system, including the olfactory bulb (Lu et al., 2005). Additionally, hypoxia has been shown to impair vision in a species of snapper owing to the inability to meet the high energetic demands of the eye under hypoxic conditions (Robinson et al., 2013). Similarly, hypoxia has been shown to impair functioning of the auditory nerve in goldfish, possibly because of an impairment of neurotransmitter release (Fay and Ream, 1992). Therefore, it is possible that external hypoxic conditions may impair olfactory sensory neurons in a similar way, but this has not been previously tested.

The functional units of the olfactory system are the olfactory sensory neurons (OSNs) located in the olfactory epithelium. There are several different forms of OSNs, classified by their cellular projections and structures, and they include microvillous, ciliated and crypt cells (Kermen et al., 2013). These neurons are in direct contact with the external environment, and the odorant receptors are found in their cilia and microvilli. These receptors bind to odorants, activating G-proteins, which in turn activate adenylyl cyclase to increase intracellular cAMP concentrations using ATP as a substrate (Olivares and Schmachtenberg, 2019). This increase in cAMP activates ion channels, allowing an influx of ions across the membrane to depolarize the neuron, eventually evoking action potentials that are then sent to the olfactory bulbs for processing (Kermen et al., 2013; Olivares and Schmachtenberg, 2019). This process is reliant on ATP availability, which may be limited by hypoxia. Because the olfactory epithelium is in direct contact with the external environment, and is avascular, it is reasonable to assume that environmental conditions, such as hypoxia, can affect its functioning. Previously, it has been shown that other external conditions, such as increased CO_2_, can directly impair the olfactory sensitivity of fishes (Porteus et al., 2018; Velez et al., 2021).

The purpose of this experiment was to determine whether exposing the olfactory epithelia to external hypoxia affects the olfactory sensitivity of marine fish. Specifically, we tested the effects of hypoxia in the external environment while the fish itself remained normoxic. For this experiment, we chose the gilt-head seabream (*Sparus aurata*) as it is a coastal species found in areas where hypoxia is becoming more prevalent. We used three amino acids as odorants: l-cysteine, l-arginine and l-leucine. These odorants are known to elicit a strong olfactory response in many species of fish (Costa et al., 2022; Porteus et al., 2018; Velez et al., 2019). All the odorants were tested at five different concentrations at three levels of oxygen – normoxia, mild and moderate hypoxia (20, 12.5 and 5.7 kPa O_2_, respectively) – while the fish was normoxemic. We hypothesized that hypoxia would affect the olfactory sensitivity of the seabream and decrease its response to the odorants, potentially owing to a limited oxygen supply to the sensory neurons from the outside environment. We predicted that the recorded amplitude of electro-olfactogram responses would be lower with increasing severity of hypoxia.

## MATERIALS AND METHODS

Gilt-head seabream (*Sparus aurata* Linnaeus 1758; *n*=8) with a fork length between 17 and 21 cm, 200–300 g were used. Animal maintenance and experimentation were carried out in certified experimental facilities and followed Portuguese national legislation (DL 113/2013) under a ‘group-1’ licence by the Veterinary General Directorate, Ministry of Agriculture, Rural Development and Fisheries of Portugal. Gilt-head seabream were obtained from a commercial supplier (Maresa - Mariscos de Esteros, SA, Huelva, Spain) and maintained at the experimental station of Ramalhete (University of Algarve, Portugal) in 1000 litre tanks with continuously running natural seawater, under natural photoperiod and temperature and fed daily with commercial pellets (Sparos, Olhão, Portugal) (Costa et al., 2022).

For these experiments, fish were anaesthetized in seawater using 300 mg l^−1^ MS-222, buffered with sodium bicarbonate, until they no longer responded to physical stimulus. Fish were then injected intramuscularly with gallamine triethiodide (1 mg/100 g of body mass) to limit movement. Fish were then placed upright onto a padded V-support, and aerated seawater with 150 mg l^−1^ MS-222 was passed over the gills on a recycling circuit. Electrodes were made from borosilicate glass (G150TF-4, Warner Instruments, www.warneronline.com) pulled to a tip (approximately 0.2 mm diameter) and filled with 4% agar in 3 mol l^−1^ NaCl and bridged to solid-state electronics through 3 mol l^−1^ KCl and an Ag/AgCl pellet. The recording electrode was placed close to the olfactory epithelium in a place that resulted in the maximum amplitude response to 10^−3^ mol l^−1^ l-cysteine, which was close to the raphe between two of the larger rear lamellae. The reference electrode was placed onto the skin just outside of the nare. The fish were grounded by a copper wire that was inserted into the side of the fish, by the base of the tail. The gills were irrigated with aerated seawater (20 kPa O_2_) with 150 mg l^−1^ MS-222 during the duration of the experiment. Therefore, only the water running over the olfactory epithelium was hypoxic (and contained the odorants), but the fish remained normoxemic. Electro-olfactograms (EOGs) are recordings of signals that are direct current field potentials generated as the summed potentials of OSNs at the surface of the olfactory epithelium (Scott and Scott-Johnson, 2002). Recordings were initially amplified through a DC pre-amplifier and headstage (NL102, Digitimer Ltd, Welwyn Garden City, UK). Signals were then filtered above 30 Hz (NL125, Digitimer) and were amplified to a final amplification of ×2000 (NL106, Digitimer). Finally, the signals were digitized (Digidata 1440A, Molecular Devices, San Jose, CA, USA) and recorded to the software Axoscope (ver.11.6, Molecular Devices). At the end of the experiment, fish were euthanized by a sharp direct blow to the head followed with pithing under anaesthesia.

Odorants were made from frozen 10^−2^ mol l^−1^ aliquots of l-cysteine, l-arginine or l-leucine, and serially diluted to the final five desired concentrations: 10^−7^, 10^−6^, 10^−5^, 10^−4^ and 10^−3^ mol l^−1^. Additionally, each odorant was diluted in water of three different oxygen pressures, 20 kPa (normoxia), 12.5 kPa (mild hypoxia) and 5.7 kPa (moderate hypoxia). Hypoxia was achieved by bubbling gaseous nitrogen into 1 liter of natural seawater, and oxygen was measured directly during the bubbling using a handheld dissolved oxygen meter (Hanna Instruments, HI 98198). The bottle then was quickly and tightly sealed until it was time to dilute the odorants. Once the odorants were diluted, the vials were also quickly and tightly sealed, to ensure they remained hypoxic. Each odorant was tested in a random order, but the order of oxygen levels tested was always normoxic, mild and then moderate hypoxia, in case hypoxia exposure caused irreversible effects. The amplitude of responses to all odorants was normalized against the response to 10^−3^ mol l^−1^ l-cystiene in normoxia, as it provides the strongest and most consistent response of all three odorants. Multiple recordings of the response to 10^−3^ mol l^−1^ l-cysteine in normoxia were taken throughout each experiment between treatments for normalization. After the last odorant in moderate hypoxia was tested, normoxic water was flushed over the olfactory epithelium for 5 min, and the amplitude of the recorded response to 10^−3^ mol l^−1^ l-cysteine in normoxia was tested again, to determine whether any decrease observed was reversible, and that the olfactory epithelium was still responding as expected.

All statistical analyses were conducted in R (version 4.3.3). To ensure that all data met the assumptions of normality and equal variance, a Shapiro–Wilk test and Bartlett's test were conducted, respectively. To compare the effects of hypoxia on the response to an odorant, normalized data were used in a two-way repeated-measures ANOVA analysis, with both odorant concentration and oxygen level as factors, accounting for individual fish being measured multiple times. The detection threshold, the lowest odorant concentration that produced a detectable response (above baseline), was determined by plotting the amplitude of the EOG response for each fish versus the odorant concentration and plotting a line of best fit through the data. The detection threshold was defined as the *x*-value where *y* is greater than zero (the *y*-intercept). Difference in the slope of the line of best fit and detection threshold were tested with a one-way ANOVA.

## RESULTS AND DISCUSSION

**Fig. 1.**
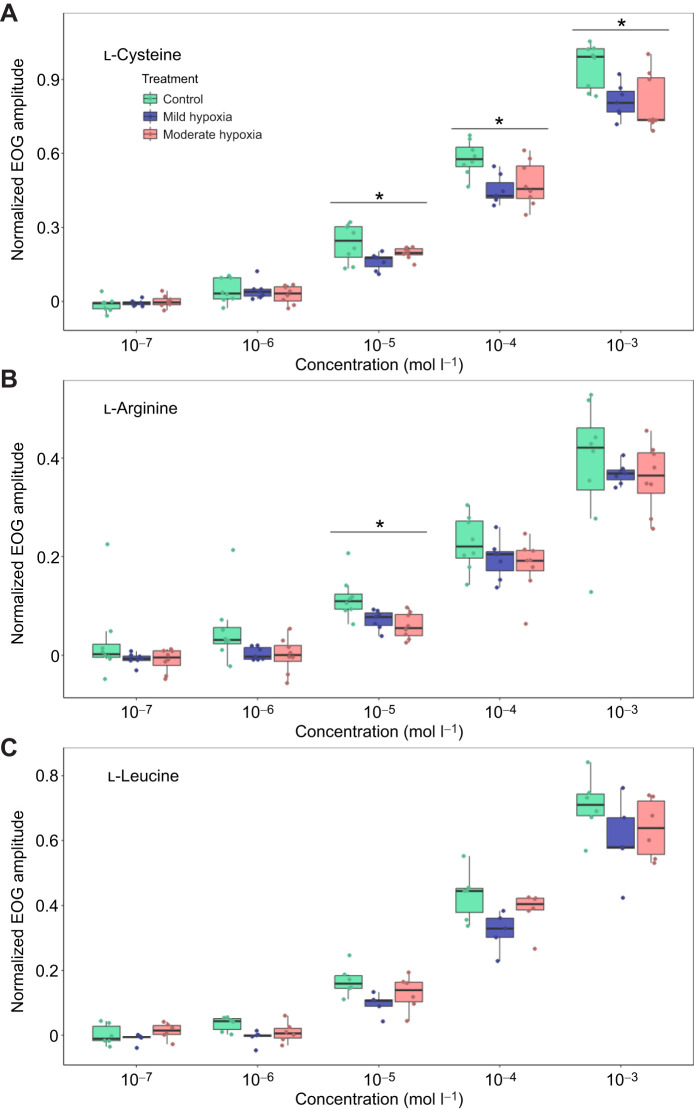
**The effects of hypoxia on the electro-olfactogram response of the olfactory epithelium of gilt-head seabream to different environmentally relevant concentrations of three amino acids.** (A) Seabream (*n*=8) showed a significant decrease in sensitivity to l-cysteine under hypoxic conditions (*P*<0.001). The response was significantly lower compared with the control (20 kPa O_2_) in both mild hypoxia (12.5 kPa O_2_; *P*<0.001) and moderate hypoxia (5.7 kPa O_2_; *P*<0.0001). There was no significant difference between hypoxia treatments (*P*=0.55). These differences were only observed at the three highest concentrations (10^−5^, 10^−4^ and 10^−3^ mol l^−1^). (B) Seabream (*n*=8) showed a significant decrease in sensitivity to l-arginine under hypoxic conditions (*P*<0.001). The response was significantly lower in both mild (*P*=0.02) and moderate hypoxia (*P*=0.003) compared with normoxia, but this was only observed at 10^−5^mol l^−1^ l-arginine. There was no significant difference between hypoxia treatments (*P*=0.83). (C) Seabream (*n*=7) experienced no difference in sensitivity to l-leucine under hypoxic conditions (*P*=0.09). Sets of boxes marked with an asterisk indicate where there is a significant (*P*<0.05) difference in the amplitude of the response between the control and hypoxic treatments.

Our aim for the present study was to determine whether external hypoxia can impair olfaction in fish. Our results indicate that hypoxia can decrease the olfactory sensitivity of juvenile seabream to some odorants. The effect of hypoxia on olfaction was different depending on the odorants tested, with the strongest effect found on l-cysteine (*n*=8; Fig. 1A). There was a significant effect of oxygen level (*F*_2,12_=10.02, *P*<0.001) and odorant concentration (*F*_2,10_=639, *P*<0.0001). The interaction between oxygen level and l-cysteine concentration was also significant (*F*_8,48_=5.30, *P*<0.0001). *Post hoc* analysis indicated that between control and both hypoxic treatments there was a significant decrease in the response observed in the highest three odorant concentrations (10^−5^ mol l^−1^: *F*_2,12_=6.05, *P*=0.042; 10^−4^ mol l^−1^: *F*_2,12_=9.81, *P*=0.003; 10^−3^ mol l^−1^: *F*_2,12_=6.33, *P*=0.013), but there was no significant difference between oxygen concentrations at the lower odorant concentrations (10^−6^ mol l^−1^: *F*_2,12_=0.30, *P*=0.75; 10^−7^ mol l^−1^: *F*_2,12_=0.11, *P*=0.90). As these lower concentrations are approaching the estimated threshold concentration of detection under ideal conditions in this species (Hubbard et al., 2003), it is not surprising that their responses under hypoxia are not significantly different. It was surprising to find that mild hypoxia at roughly 60% oxygen saturation was enough to impair the olfactory response. Generally, stronger physiological impairments only begin to occur closer to the critical oxygen tension of the fish (Robinson et al., 2013; Ultsch and Regan, 2019). The mild level of hypoxia used in this experiment was higher than the level of hypoxia that is known to cause physiological impairment in this species (63% versus 35% O_2_ saturation; Remen et al., 2015).

Interestingly, the effect of oxygen concentration on the response to l-arginine was different to that of l-cysteine. There was a significant effect of odorant concentration (*n*=8, *F*_2,12_=152.34, *P*<0.0001; Fig. 1B), and an effect of oxygen concentration (*F*_2,12_=10.02, *P*<0.0001) on the olfactory response to l-arginine, but there was no significant interaction (*F*_8,48_=0.12, *P*=0.99). *Post hoc* analysis showed a significant difference between oxygen level only at 10^−5^ mol l^−1^ l-arginine (*n*=8, *F*_2,12_=12.3, *P*=0.001). In l-leucine, there was no significant effect of oxygen level on the olfactory response (*n*=8, *F*_2,12_=3.28, *P*=0.09; Fig. 1C), but there was a significant effect of odorant concentration (*F*_4,16_=528.19, *P*<0.0001) on the EOG response. Based on the amplitude of the EOGs, the responses to l-leucine and l-arginine were smaller in all conditions compared with l-cysteine (Fig. 1), which is similar to what has been observed in this species previously (Hubbard et al., 2003). All three of these amino acids have different structures, and presumably bind to different olfactory receptors which, in turn, are located on different OSNs. As we did not observe similar results between odorants, this indicates that different OSNs are affected by hypoxia to different extents.

**Fig. 2.**
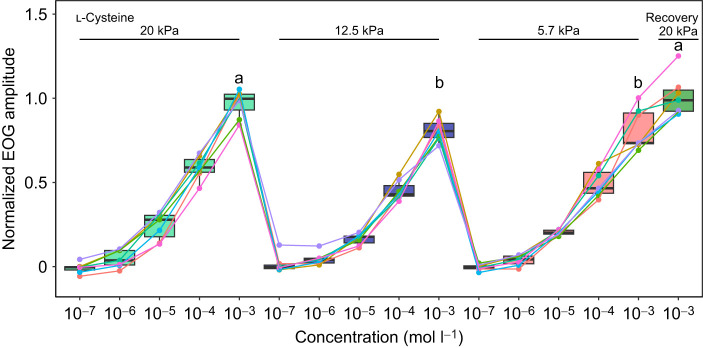
**Repeated responses of seabream olfactory epithelium to l-cysteine in different *P*_O_2__ tested in series.** There was a significant effect of oxygen concentration on the amplitude of the response to l-cysteine (*P*<0.0001). The response was 16% lower in the mild hypoxia treatment (12.5 kPa O_2_) and 17% lower in the moderate hypoxia treatment (5.7 kPa O_2_) at the highest concentration (10^−3^ mol l^−1^) compared with the control (20 kPa O_2_). After the olfactory epithelium was rinsed with normoxic water for 5 min, the response recovered and was not statistically distinct from the original normoxic treatment (*P*=0.87). The results are presented in the chronological order in which they were tested. Each individual seabream is represented with a different colour point and line. Boxes with different letters indicate a significant difference in the amplitude of the response recorded at the highest concentration of l-cysteine (10^−3^ mol l^−1^).

At the highest concentration of l-cysteine, there was an average of a 16% decrease in the amplitude of the response in the mild hypoxia group, and a 17% decrease in the moderate hypoxia group compared with the control group, indicating a moderate effect of environmental hypoxia on olfactory detection. It is possible that the hypoxic conditions are limiting the production of ATP within these cells. As the microvilli and cilia, which contain the receptors and transduction machinery, are in direct contact with the external environment, and the olfactory epithelium is avascular, it is likely that – within fish – the olfactory epithelium takes up oxygen directly from the water. It has previously been shown that in rat olfactory epithelia, OSNs can take up oxygen through their external processes, such as cilia (Bigdai et al., 2019). These external processes, such as cilia or microvilli, are the sites of odorant receptors in some classes of OSNs. Additionally, in mammals at least, the cilia of OSNs get ATP from two sources: the production of ATP in mitochondria within the main body of the cell (i.e. the soma), and glycolytic production of ATP in the apical point of the cell (i.e. the dendritic knob), where the cilia are anchored (Villar et al., 2021). It is possible that the production of ATP is therefore limited with less oxygen available from the environment, leading to the diminished response, although there is still some ATP available from glycolytic processes, explaining a reduced response rather than a complete absence of one. Furthermore, this reduction in available ATP may not affect all OSNs similarly, where some may be able to illicit a response, whereas others are not, resulting in the lower observed amplitude in the recorded response. The fact that the effect was completely reversible when normoxic water was tested at the end of the experiment Fig. 2 also suggests that limited oxygen for ATP production may be the cause of the impairment.

The detection threshold is defined as the concentration below which odorants are not detected. Although there were reductions in the amplitude of response to l-cysteine under hypoxic conditions, there was no significant difference in detection threshold between normoxia and the two levels of hypoxia (*n*=8, *F*_2,18_=0.83, *P*=0.45; Table 1). This was also the case for l-arginine; *n*=8, *F*_2,18_=1.69, *P*=0.21). However, for l-leucine, there was a marginally non-significant difference in the detection threshold between oxygen concentrations (*n*=8, *F*_2,18_=3.74, *P*=0.05). The detection threshold was significantly higher in mild hypoxia compared with the control (*P*=0.04), but in moderate hypoxia it was not significantly different from the control (*P*=0.44) nor from the mild hypoxia treatment (*P*=0.31). This is different than what has previously been found in fish exposed to ocean acidification. When seabass were exposed to high CO_2_, the detection threshold increased between 2- and 5-fold, depending on the odorant tested (Porteus et al., 2018). This increase in detection threshold under high CO_2_ is likely caused by the change in odorant and/or receptor protonation decreasing binding affinity with the OSNs (Porteus et al., 2021). Hypoxia would not change the structural composition of odorants, so the overall binding affinity would be less affected, limiting the effects on detection threshold. With the changes observed of the normalized response, and the limited effects on detection threshold, the effect hypoxia has on olfaction likely comes from the energetic demands of the OSNs being unable to be met.

**Table 1.** The slope of response curves and detection thresholds of different odorants at different oxygen levels recorded in the olfactory epithelium of gilt-head seabream

Odorant	Treatment	Slope (±s.e.m.)	Detection threshold (**mol l^−1^**) (±s.e.m.)
l-cysteine	Control	0.307±0.012^a^	2.10×10^−06^±0.13
	Mild hypoxia	0.259±0.012^b^	1.24×10^−06^±0.04
	Moderate hypoxia	0.261±0.014^b^	1.19×10^−06^±0.05
l-arginine	Control	0.131±0.010	1.09×10^−06^±0.08
	Mild hypoxia	0.122±0.003	1.62×10^−06^±0.05
	Moderate hypoxia	0.121±0.009	2.44×10^−06^±0.12
l-leucine	Control	0.228±0.011	1.10×10^−06^±0.04^a^
	Mild hypoxia	0.205±0.016	1.95×10^−06^±0.05^b^
	Moderate hypoxia	0.214±0.014	1.47×10^−06^±0.09^a,b^

Different superscript letters indicate significant differences between treatments.

To our knowledge, this is the first study to examine whether hypoxia can negatively affect the olfactory system in fish through electrophysiological measurements. We found evidence that hypoxia broadly impairs olfaction with slight differences between odorants tested. This means that, even under mild hypoxia, the ability of these fish to find food may be impaired, leading to a potential decline in fitness. It is possible there may be species-specific differences based on hypoxia tolerance, and it is likely that a hypoxia-tolerant species would not experience such an impairment, and would serve as an interesting continuation of this research. Alternatively, long-term exposure to hypoxia may result in plastic changes occurring that may impair the olfactory system further, which may be more ecologically relevant. This may also result in an upregulation of anaerobic pathways within the OSNs. However, the fact that hypoxia did impair olfaction in this species does have concerning implications in how many species may be negatively affected by yet another environmental consequence of the Anthropocene.
